# Supporting family carers in general practice: a scoping review of clinical guidelines and recommendations

**DOI:** 10.1186/s12875-023-02188-1

**Published:** 2023-11-06

**Authors:** Mary Cronin, Kathy McLoughlin, Tony Foley, Sinéad McGilloway

**Affiliations:** 1 Centre for Mental Health and Community Research, Department of Psychology and Social Sciences Institute, Maynooth University, Maynooth, Ireland; 2 Health and Social Care, Chevron College, Wexford, Ireland; 3 Department of General Practice, University College Cork, Cork, Ireland

**Keywords:** Family Carer, General Practice, GP, Clinical guidelines, Primary care, Scoping review

## Abstract

**Background:**

Increasing numbers of family carers are providing informal care in community settings. This creates a number of challenges because family carers are at risk of poor physical and psychological health outcomes, with consequences both for themselves and those for whom they provide care. General Practitioners (GPs), who play a central role in community-based care, are ideally positioned to identify, assess, and signpost carers to supports. However, there is a significant gap in the literature in respect of appropriate guidance and resources to support them in this role.

**Methods:**

A scoping review was undertaken to examine clinical guidelines and recommendations for GPs to support them in their role with family carers. This involved a multidisciplinary team, in line with Arksey & O’Malley’s framework, and entailed searches of ten peer-reviewed databases and grey literature between September-November 2020.

**Results:**

The searches yielded a total of 4,651 English language papers, 35 of which met the criteria for inclusion after removing duplicates, screening titles and abstracts, and performing full-text readings. Ten papers focused on resources/guidelines for GPs, twenty were research papers, three were review papers, one was a framework of quality markers for carer support, and one was an editorial. Data synthesis indicated that nine (90%) of the guidelines included some elements relating to the identification, assessment, and/or signposting of carers. Key strategies for identifying carers suggest that a whole practice approach is optimal, incorporating a role for the GP, practice staff, and for the use of appropriate supporting documentation. Important knowledge gaps were highlighted in respect of appropriate clinical assessment and evidence-based signposting pathways.

**Conclusion:**

Our review addresses a significant gap in the literature by providing an important synthesis of current available evidence on clinical guidelines for GPs in supporting family carers, including strategies for identification, options for assessment and potential referral/signposting routes. However, there is a need for greater transparency of the existing evidence base as well as much more research to evaluate the effectiveness and increase the routine utilisation, of clinical guidelines in primary care.

**Supplementary Information:**

The online version contains supplementary material available at 10.1186/s12875-023-02188-1.

## Background

The provision of care in the community has attracted increasing concern in recent years due to the growth in ageing populations, lower birth rates [12], shifting societal demographics [15], and changes in health care delivery [16]. However, care for many vulnerable citizens is provided largely by family members or loved ones, who are described as ‘family carers’ or ‘informal carers’. For example, in Europe alone, it is estimated that 10–25% of care in the community is provided by family carers [17]. In Ireland, the support/labour provided by family carers saves the state an estimated €20 billion in care costs annually [19].

A wealth of evidence indicates that these carers report poorer physical and mental health outcomes than the general population [9, 10, 18, 19]. For example, a recent study by Gallagher et al. found that carers had a 33% increased risk of future illness or disability when compared to non-carer controls [20]. Crucially, this impact on carer health appeared to be present even beyond the end of the caring role. Furthermore, carers typically report higher levels of psychological distress [21] than non-carers [22]. Despite these psychological and physical impacts, robust systems to support those who provide care continue to be ad hoc, inconsistent, or absent [23].

A growing body of evidence suggests a number of barriers to the provision of appropriate systems for supporting family carers [23]. The identification of carers, in the first instance, can be challenging, as many carers do not identify with the term ‘carer’ but, instead, identify with the relationship to the person for whom they are providing care [6]. Furthermore, health care professionals (HCPs) are not always aware of who is providing the care and, even when they are, evidence suggests they are reluctant to raise the question, as they are unsure of their role in this regard [21, 24]. Thus, even when carers are identified, there is a lack of clear direction regarding how their needs can be best assessed and to where they can be referred or signposted for support [21].

The National Institute for Care Excellence (NICE) suggests that health and social care practitioners should “actively seek to identify carers” [6] (p.12). Furthermore, existing literature highlights, in particular, the benefits of a role for general practitioners (GPs) in identifying and supporting family carers [25]. A number of countries or regions, such as the UK and parts of Australia and Canada, have developed guidelines for GPs in their role vis-à-vis carers [1, 5, 8], whilst other studies have examined how GPs may support specific caring roles [26]. Guidelines are often used in primary care to support and improve patient care [27], and are either produced by GP professional bodies, external agencies, or adapted from national guidelines [28]. However, no guidelines to support GPs in their role with family carers are, as yet, available in many countries across the world despite the fact that evidence-based guidelines can be an important resource for GPs in a clinical setting [28]. The barriers and facilitators to supporting carers in general practice have been widely researched and identified [29], but there is still little published literature regarding the provision of appropriate and effective clinical guidelines for the support of family carers.

The current study was conducted as part of a larger project that investigated how family carers in Ireland are supported in health care settings, with a particular focus on general practice. The aims of the study reported here, were: (1) to identify and critically review the existing national and international guidelines, practice standards, procedures, and/or other literature relevant to the development, implementation, and evaluation of clinical practice guidance for GPs, in order to assist them to identify, assess, and signpost family carers in general practice; (2) identify examples of good practice that have been demonstrated to support the identification, assessment, and referral of family carers in general practice; and (3) to help inform the development of guidelines and accompanying education and audit resources for use by GPs in Ireland. The specific research questions that guided the study were:

1. What clinical guidance is available to GPs to support carer identification and assessment?
2. What guidance is available to enable GPs to signpost family carers to relevant services/supports?
3. What processes are in place (if any) to evaluate the effectiveness of the above guidance?
4. What resources are available to support GPs in the identification, assessment, and referral process of family carers?
5. What is the level of evidence available for clinical guidance on supporting family carers?

We used a scoping review methodology that allowed us to explore or ‘scope’ the broad topic of clinical guidelines in respect of family carers, in both peer-reviewed and grey literature. Scoping studies are particularly useful in exploring areas that have not been comprehensively reviewed and where the evidence is emerging [30], or based on a broad range of study designs and methodologies [31]. We expected that few, if any, randomised controlled trials (RCTs) would be available and that much of the literature would be based on guidance produced by professional GP bodies and carer support agencies, as well as other sources of grey literature.

## Method

The scoping review method used here was in line with the original guidelines proposed by Arksey and O’Malley [31], whilst also incorporating more recent revisions and suggestions [32, 33]. This involved a six-step process including: (1) identifying the research question; (2) identifying relevant studies; (3) selecting studies; (4) charting the data; (5) collating, summarising and reporting the results; and (6) consulting with key stakeholders. We used the Preferred Reporting Items for Systematic Reviews and Meta-Analyses extension for Scoping Reviews (PRISMA-ScR) guidelines in reporting the findings [34] (see Additional File 1). We did not publish a protocol for this review.

### Search terms and databases

The PICOS (Population, Intervention, Comparison, Outcome, Setting) framework was used to formulate the research question and to identify appropriate and relevant search terms [35]. A full outline of the PICOS components is provided in Additional File 2. The search terms were formulated by the lead (MC) and second author (KMcL), and circulated to the wider team for review before being finalised. Searches were carried out between September and November, 2020. We included studies relating to general practice or primary care and any interventions/guidance that supported the identification, assessment, or signposting of family carers in these settings. We excluded studies related to paid carers such as Health Care Assistants (HCAs) and hospital or nursing home settings. The databases of peer-reviewed and grey literature searched were: CINAHL, Medline, PsycINFO, Lenus.ie, Google – first 200 results [69, 70], OpenGrey, NICE, Cochrane, and Kingsfund. A search was also undertaken of GP professional bodies and government websites from countries considered by the OECD [36] to be proactive in carer assessment (e.g. UK, Sweden, and Australia), as well as those where larger numbers of research papers on the topic were generated, such as Canada and USA. The databases and other websites searched (and including GP professional bodies), were selected in consultation with the full research team. The full search strategy for Medline, including medical subject headings (MeSH), is available in Additional File 2. Papers from the previous 10 years (Jan 2010 – Oct 2020) were included in order to gain up-to-date clinical guidance. All included papers were in the English language due to time and funding constraints.

### Identifying relevant studies and study selection

The first author (MC) conducted searches on databases, grey literature and professional bodies, as well as hand searching of reference lists of retrieved papers, while the second author (KMcL) searched the CINAHL database. Searches were limited to title and abstract. Papers were imported into Mendeley for initial data management purposes such as de-duplication, and titles and abstracts were screened for removal of obviously irrelevant papers. The full texts of included papers were retrieved and imported into Rayyan software for full text review by both MC and KMcL. In the event of any disagreement, another member of the multidisciplinary research team (TF) acted as a third reviewer. This team approach to data extraction was used to ensure rigor [32]. Furthermore, although we were expecting a low level of evidence (LoE), we decided to rate the included studies using the seven hierarchical levels of evidence outlined by Ackley et al. [37] (see Table 1).

**Table 1:** Level of evidence rating scheme. Based on: Ackley BJ, Swan BA, Ladwig G, & Tucker S. Evidence-based nursing care guidelines: Medical-surgical interventions. St. Louis, MO: Mosby Elsevier. 2008;7

Level of evidence (LOE)	Description
Level I	Evidence from a systematic review or meta-analysis of all relevant RCTs (randomized controlled trial) or evidence-based clinical practice guidelines based on systematic reviews of RCTs or three or more RCTs of good quality that have similar results.
Level II	Evidence obtained from at least one well-designed RCT (e.g. large multi-site RCT).
Level III	Evidence obtained from well-designed controlled trials without randomization (i.e. quasi-experimental).
Level IV	Evidence from well-designed case-control or cohort studies.
Level V	Evidence from systematic reviews of descriptive and qualitative studies (meta-synthesis).
Level VI	Evidence from a single descriptive or qualitative study.
Level VII	Evidence from the opinion of authorities and/or reports of expert committees.

### Charting and data synthesis

Data charting involves mapping out the data according to key issues and themes [31]. As recommended by Levac et al. [32], we completed an additional step to charting which involved two reviewers (MC & KMcL) independently reviewing the first five to ten papers using the charting form and then consulting to see if our approach was consistent and in line with the core research question. This ‘trial charting exercise’, followed by consultation, was very helpful in ensuring the richness of the data [33]. The first author (MC) developed a draft form to encompass a range of items including author and publication details, as well as: (1) aims/ objectives; (2) study population and sample size; (3) setting (i.e. primary care or general practice); (4) identification; (5) assessment; (6) signposting; (7) consultation resources; and (8) level of evidence. The first and second authors (MC and KMcL) then piloted the form, as recommended [32], resulting in the inclusion of one additional item (i.e. documentation).

### Consultation exercise with stakeholders

Arksey & O’Malley [31] suggest that stakeholder consultation should be an optional step in a scoping review, while Levac et al. [32] and Daudt et al. [33] go farther by recommending it as a requirement; interestingly, Daudt et al. [33] argue that several stakeholders should be included on the review team in order to enhance the consultation process. Thus, our multidisciplinary research team included a number of key stakeholders in the form of a GP (TF), and a psychologist from a national carer support organisation (KMcL) to ensure relevance of the study to clinical practice; the first author (MC) is also a carer with over 20 years’ experience in that role while the last author (SMcG) is a senior academic with considerable experience in conducting reviews. However, as the results of this scoping review were intended to be applied to inform the development of clinical guidelines (also known as ‘clinical practice points’), we felt it was important to include the voice of carers as primary stakeholders. Therefore, a consultation exercise was conducted with a panel of carers (N = 5) from a number of diverse caring roles (e.g. a son caring for his father, wife caring for husband, etc.). A draft of the ‘practice points’ was presented to the carers for comment during this exercise, with their input incorporated into the final set of clinical practice points.

## Results

**Fig. 1:**
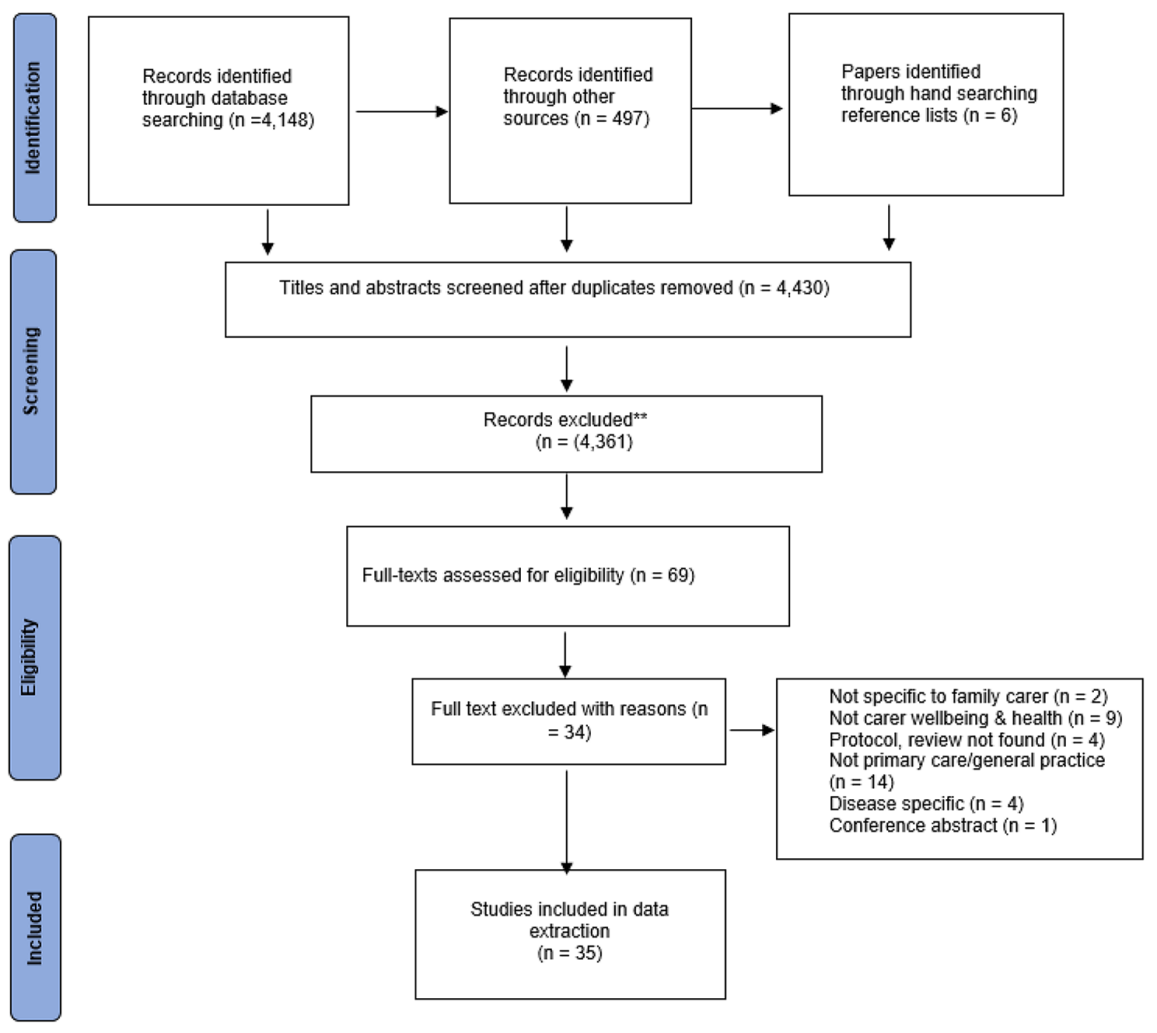
PRISMA Diagram of literature search

A total of 4651 papers were retrieved, 4,430 (95%) of which were included in title and abstract screening following deduplication. Sixty-nine papers met the eligibility criteria for full text review, 35 of which were selected for inclusion in the review (Fig. 1). Further details relating to the numbers of papers per database and specific search dates are included in Additional File 3.

### Characteristics of included studies

Ten papers were categorised as resources or guidelines designed for GPs to support them in their role with family carers, twenty were based on peer-reviewed research (12 qualitative, 5 quantitative, 1 study protocol, 1 RCT and 1 systematic review), three were review papers (2 narrative reviews, 1 literature review), one was a quality marker indicator for carer support, and one was an editorial paper. Whilst four of the included studies focused solely on carers, 12 involved the recruitment of participants from other sources including GPs, general practice staff, other health care staff and policy makers (See Table 2). Sixteen of the included research studies focused on primary care/general practice settings, with carer participants providing support to family members with issues ranging from advanced cancer and palliative care to older person care, stroke and dementia. Sample sizes ranged from 19 to 40 for 12 of the included qualitative studies, with the exception of one study with a sample size of 70 recruited from a diverse group of stakeholders. The largest sample size of all the included studies (N > 800) was reported for a piece of work that explored the components of the Family Strain Questionnaire with a view to developing a shorter psychometrically robust version. Most included studies were from the UK (12), with the remainder based in the USA (5), Canada (3), Australia (3), Germany (1), Norway (1), Italy (1), and Ireland (1).

**Table 2:** Characteristics of included studies

Study/Guidelines	Aim	Study Design	Country	Participants and sample size	Level of Evidence**
Burridge, Mitchell et al. (2011)	To explore the views of lay caregivers and health professionals about the way lay caregivers’ health concerns are raised by their GP?	Qualitative: Semi-structured interviews	Australia	Cancer Caregivers (n = 6) Health professionals (n = 19).	VI
Burridge, Mitchell et al. (2017)	Explores carers and GPs’ views regarding the acceptability and usefulness of the NAT-C for helping carers to address their own health concerns.	Qualitative: Semi-structured interviews	Australia	Cancer caregivers (n = 11) and GPs n = 5).	VI
Carduff et al. (2014)	To identify barriers to and explore strategies for identifying carers in primary care. Particularly self-identifying as carer and identifying those caring in end of life.	Triangulated data: Lit review, workshop and focus groups	UK (Scotland)	Lit review (n = 50 papers), Researcher workshop (n = 70), Focus groups carers (n = 15), health professionals (n = 8)	VI
Carduff, Jarvis et al. (2016)	To develop, pilot, and evaluate a new model of identifying, assessing, and supporting unpaid carers of people with palliative care needs.	Feasibility study, qualitative evaluation interviews	UK (Scotland)	Carers of terminally ill in 4 GP practices. (n = 81) received carer pack, (n = 25) returned CSNAT form, (n = 11) took part in follow-up interviews.	VI
Family caregiver alliance (2012)	To provide practitioners with a wide range of measures from which they may generate assessment instruments appropriate and applicable to their practice setting, and beneficial for care planning.	Assessment measures resources inventory	USA	N/A	N/A
Fisher et al. (2020)	To identify barriers and facilitators faced by HCPs in supporting FCGs, as well as knowledge, skills and attitudes needed by HCPs, to provide comprehensive services to FCGs.	Qualitative – symposium to gather perspectives of FCG’s, HCP’s and stakeholders	Canada	N = 40, FCGs n = 8 (Caregivers of seniors), frontline HCPs n = 6, managers n = 3, senior services organizers n = 3, non-government organizations leaders n = 6, academics n = 11 policy makers n = 3.	VI
Greenwood et al. (2010)	Investigate GPs’ attitudes to carers, awareness and knowledge of issues facing carers and perceived barriers to supporting carers.	Post-training questionnaire survey of GPs	UK	Practice managers and receptionists) n = 33	VI
Greenwood et al. (2011)	Explores the support stroke carers would like from general practice and reactions to a community-based support and perceptions of a general practice team on carer supports.	Qualitative study – Semi-structured interviews	UK (England)	Stroke carers (n = 13) General practice staff (n = 10) GPs from varying sized practices. N = 78	VI
Greenwood et al. (2016)	Identify, appraise, and summarize all the published evidence on general practice-based interventions to support carers of people with stroke or dementia.	Systematic Review	UK (England)	4 included studies – all dementia carers	I
Jiwa et al. (2010)	To develop an innovation to be tested in a formal clinical trial in Australian general practice (p.10). Pilot testing of NAT-C prior to RCT.	Complex intervention using actor patients	Australia	GPs (n = 6) Actor patients (n = 6), 34 recorded consultations.	VI
Jones et al. (2012)	Inform the Department of Health about the impact and efficacy of the pilot workshop programme in increasing the participants’ knowledge and awareness about carers and how they might be assisted.	Questionnaire evaluation pre-workshop, post-workshop and 3 months post-workshop	UK	GPs (n = 95), clinical primary care workers (practice nurses, HCA’s), community matrons (n = 25), non-clinical primary care workers	VI
Kingston University (2010)	Evaluate six pilot workshops across England as part of the National Education Programme for Supporting Carers in General Practice, organized by the Royal College of General Practitioners and the Princess Royal Trust for Carers.	Evaluation of RCGP pilot training for GPs. Questionnaires: preworkshop, end of workshop and post three months	UK	Six pilot workshops, total participants n = 192, total participants working in primary care n = 153. Workshops delivered by 2 GPs and 1 former carer	VI
Katja Krug et al. (2018)	Increase the knowledge about challenges in general practice for patients, lay carers, and professionals in end-of-life (EoL) care.	Qualitative – focus groups	Germany	GPs (n = 12), medical assistants (N = 7) – with a special interest in palliative care.	VI
Mitchell et al. (2010)	To assess the efficacy of the systematic utilization of a GP Toolkit in reducing caregivers’ reported number and level of unmet needs AND Evaluate the acceptability of the intervention for GPs and caregivers.	Study protocol for RCT		GPs and caregivers (approx. 400 caregivers and 330 GPs to complete the study)	N/A
Mitchell et al. (2013)	To assess the hypothesis that the efficacy of a GP-based intervention incorporating a carer-reported needs checklist and a supporting GP Toolkit of resources, reduces the reported number and intensity of unmet carer needs, compared with usual care.	RCT - general practice	Australia	Carers of people with advanced cancer(N = 392)	II
National Health Service (NHS) England, patient experience team (2016)	Developing an integrated approach to the identification, assessment, and support of Carers and their families across health and social care.	A resource to help promote working together between Adult social care services, NHS commissioners and providers, and third-sector organizations	UK	N/A	N/A
National Institute for Health and Care Excellence (NICE), (2020)	Guideline providing action-orientated recommendations for good practice, aimed at improving outcomes for adult carers.	Recommendations for health and social care practitioners in supporting Adult Carers.	UK	N/A	N/A
O’Connor C. (2011)	Assess the role of Ireland’s general practitioners in caring for dementia carers.	Literature Review	Ireland	Dementia caregivers and general practitioners, general practice-based studies	N/A
Onwumere (2016)	Article in British Journal of General Practice discussing how GPs are in a unique position to support individuals with psychosis and carers in general practice.	Editorial	UK	General practice audience	VII
Parmar et al. (2020)	(1) To review stakeholder engagement process that led to the development of the competencies, (2) describe the process used to identify the competency domains, (3) report on the modified Delphi process used to validate the domain indicators, and (4) introduce the competency framework.	Multilevel interdisciplinary stakeholder co-design to develop a competency framework	Canada	Expert panel of Stakeholders (n = 50) included family caregivers, health care leaders, not-for-profit social care leaders, health professionals, front-line health care providers, policymakers and policy influencers, national and international researchers	VI
Peters et al. (2019)	To explore the views of professional stakeholders on how health services, particularly primary care, can support carers and scope for strengthening such support in England.	Qualitative - semi-structured interviews	UK	Total n = 25, (GPs n = 4, Nurse n = 4, pharmacist n = 2, consultant n = 1, phlebotomist n = 1, policy n = 5, voluntary sector n = 8, local authority n = 1, private health sector n = 3, researcher n = 1.	VI
Royal College of General Practitioners (RCGP) (2013) – in partnership with Princes Royal Trust for carers.	Guide to help GPs understand who carers are, why they need help, how to involve them in patient care, and how to support them AND Educational tool AND summary report.	Action guide for GPs and their teams	UK	N/A	N/A
Roen et al. (2019)	To explore and describe health care professionals’ (HCPs) carer support within cancer palliative care within Orkdal district.	Qualitative - focus groups	Norway	HCPs n = 21	VI
Riffin et al. (2020)	To identify current approaches to identifying carer needs and risks in primary care, To understand the benefits and barriers to implementing a standardized caregiver assessment in primary care, to derive recommendations for integrating assessment tools into primary care.	Qualitative - semi-structured interviews	USA	Primary care clinicians, staff and administrators (n = 30), Patient and family caregivers (n = 40)	VI
Robinson et al. (2010)	Addresses long-term care at home for people with dementia with a focus on psychosocial interventions, provision of information, caregiver support, behavioral and psychological symptom management and case management.	A narrative review	UK	N/A	N/A
Royal Australian College of General Practitioners (RACGP)(2019)	To support clinicians in supporting families and caregivers of older persons.	Part B of aged care clinical guide. – families and carers	Australia	N/A	N/A
RCGP Scotland (n.d.)	To support GPs in the identification, support, and signposting of carers and young carers.	GP Resource/Information leaflet	Scotland	N/A	N/A
Smith et al. (2018)	To develop and evaluate a series of workshops intended to increase confidence as it relates to communication between caregivers, care recipients and health care professionals and thereby decrease caregiver burden.	Feasibility study	USA	Caregivers (N = 16)	VI
Sunne et al. (2017)	To provide a concise review of how to care for the caregivers.	Review paper	USA	N/A	N/A
Swartz & Collins (2011, & 2019)	Summarizing caregiver care by primary care physicians and offer direction for future research – handout for carers is included.	American Family Physician article – Caregiver Care	USA	N/A	N/A
Vidotto G (2010)	To examine the properties of the Family Strain Questionnaire in the context of the Rasch model for scale construction to pave the way to develop a shortened refined version that practitioners can use routinely to screen for caregiver stress.	Development of a short form of the family strain questionnaire (FSQ). (semi-structured interview	Italy	Caregivers (n = 811) completed original FSQ, caregivers (n = 40) participated in reanalyzing the revised shorter version	VI
Doctors of BC (British Columbia, Canada)	Tool kit for doctors - how to organize your practice to support family caregivers.	Supplementary resource part of Doctors of BC policy paper “Circle of Care: Supporting Family Caregivers in BC”	Canada	N/A	N/A
Carers Trust Wales (2019)	Designed to be used by Regional Partnership Boards, Local authorities, Local Health Boards and third sector organizations in Wales to support the identification and commissioning of good services for un-paid carers.	Good practice approaches to supporting carers in wales	UK - Wales	N/A	N/A
NHS (2019)	Quality markers for supporting carers in general practice.	Quality markers	UK	N/A	N/A
Northern Sydney Local Health District (Australia)	To provide information to GP’s on the caring experience, what it means to be a carer, the impact of caring for another person, as well as how a GP can support those important partnerships in caring.	A guide for GPs and primary care teams	Australia	N/A	N/A

**Level of evidence rating assigned to studies [37]

Health-related risks for carers were mentioned in six of the included papers and referred to symptoms of psychological distress, such as anxiety and depression [10], neglect of own health due to a focus on the care recipient, or difficulty in attending appointments [14], as well as other ailments such as back injury/ pain [14], shoulder injury [8], high blood pressure [14], greater risk of stroke [38], increased mortality in older carers [38], insomnia [39], and sleep problems [3].

#### Guidance on the identification of Carers

The identification of carers is the first step toward offering support, and this was addressed in 19 of the papers, including 9 of the 10 resource/guidance documents. Identification also forms a key part of the quality markers paper by the NHS [40]. Six papers reported on studies where carer identification was a component of the findings [2, 24, 41–44]. Two review papers discussed the support of carers in general practice [45, 46], while one paper identifying the core competencies in HCP education, included the identification of carers [29]. Overall, the identification of carers in general practice/primary care was considered to be the responsibility of the whole practice.

#### A whole-practice approach to the identification of carers

**Fig. 2:**
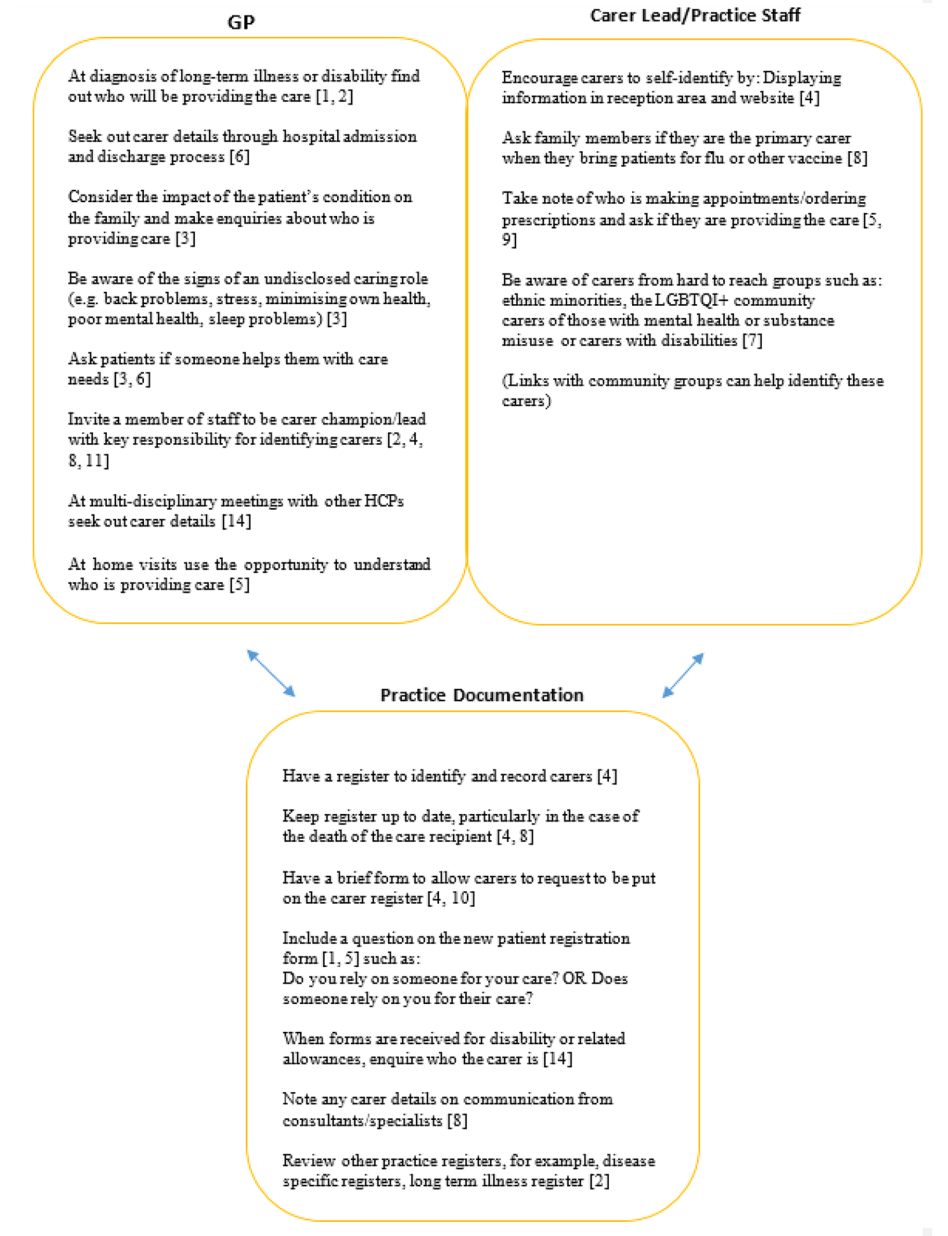
Whole practice approach to carer identification

Strategies to identify carers fell into three broad categories (see Fig. 2), including a key role for GPs, responsibilities for practice staff, and the availability and use of practice documentation. GPs may identify carers in a number of ways, including consultations with the care recipient, communication with other HCPs, pro-actively making enquiries and being alert to signs (and symptoms) of carer burden, as well as appointing a carer champion/carer lead within their practice. A number of guidelines suggest that the point of diagnosis or first appointment can be an opportunity to ascertain who will be providing most of the care or support for patients who have longer term illness/disability [1, 2]. Additionally, transitions such as the care recipient moving to adult services, or relocating to a nursing home or other form of residential care, were also highlighted as particularly stressful times when carers may need additional support [21].

#### Carer champion

The appointment of a carer champion by the GP was a recurring finding in this review [2, 11, 14, 40], defined by NICE as a “…designated member of staff who is tasked with supporting and speaking up for carers”. Carer champions can act as a key contact for carer information and advice, providing knowledgeable expert advice, as well as training other practitioners working within the service” [6] (p.32). They could have responsibility for promoting self-identification, liaising with family members, being alert to who accompanies care recipients, and being proactive about identifying carers from ‘harder- to-reach’ groups. A carer champion may be one of the clinical or administrative staff and play a significant role in carer identification [14]. One of the included papers, an RCGP educational resource for GPs and primary care teams, provides useful, more detailed guidance on the responsibilities of a carer champion, for example, maintaining the carers register, being in-practice point of contact for carers and sourcing information for carers [47] (p.32).

Documentation and record keeping are also important in supporting the identification of carers by, for example: providing a carers’ register (i.e. a list of carers in the practice that can be used to provide targeted supports such as invitations to vaccine clinics or health checks) [40], and pro-actively seeking information about carers through current practices, such as new patient registration, completion of welfare applications, communications with other HCPs, linking with in-house databases (i.e. illness specific registers), and providing routes for self-registration. The importance of keeping the carer register up-to-date was highlighted by the RCGP summary report [14], which emphasised in particular, the need to remove carers when a care recipient dies or moves to residential care. This report also usefully highlights systems that may be used to record carers in a number of exemplar practices, including coding carer status as ‘has a carer’ or ‘is a carer’ [14].

### Guidance on the assessment of carers

Twenty-two studies discussed the assessment of carers’ needs in general practice. However, just two of the ten guidelines for GPs referred to the type of assessment that may be useful. For example, the American Family Physicians resource [13], entitled ‘Caregiver Care’, refers to both the Adapted Zarit Burden Interview and the Modified Caregiver Strain Index, while ‘Doctors of British Colombia’ in their resource [5], ‘Organising your practice to support family caregivers: A toolkit for doctors’, also refer to the Adapted Zarit Burden Interview. An additional five assessment tools were indicated throughout the review including: (1) the Needs Assessment Tool – Caregivers (NAT-C) [26, 48–51]; (2) The Carer Support Needs Assessment Tool (CSNAT) [2, 52]; (3) The Adult Social Care Outcome Tool - Carer (ASCOT – Carer) [44], (4) The Carers Star (Carers Star™)The Outcomes Star for people caring for others [44] and (5) the Family Strain Questionnaire (FSQ) [53]. Notably, the Family Caregiver Alliance in the USA [54] provides a comprehensive range of measures that may be used in health and social services to assess carers across a range of domains that include physical and mental health (Sect. 4).

Although just seven assessment tools were mentioned specifically, several of the included papers discussed assessment in terms of its therapeutic and preventative effect [6, 41] and the usefulness of an assessment to facilitate communication [26, 52, 55] during a GP consultation. Other studies highlighted a need for carers to be systematically [56] and periodically [13] assessed, with interventions designed to meet their needs [56]. The structure and wording of assessments was mentioned in several papers, with suggestions that they should be brief and linked to patient outcomes, and with wording that is free from judgement about carers’ performance, or any assumption that all carers need (or want) help [55].

According to NICE guidelines, assessment can be performed by the family doctor or other health or social care team member [6]. For example, in the UK, Local Authorities (local county councils, via social care) are legislated to assess carer needs, but this can also be delegated to the voluntary sector [44]. In Wales, the North East Wales Carer Information Service offers an assessment of carers who receive support through social services. In this case, ‘Wellbeing Officers are trained to deliver the ‘what matters’ carer needs assessments, which can take up to 8 hours to complete (we unsuccessfully attempted to obtain a copy of this assessment (via email) on several occasions). As part of their Quality Markers for supporting carers [4], the National Health Service (NHS) recommend that carers have their support needs assessed and receive an integrated package of support [40, 44]. The RCGP Scotland also affirms that carers have a legal right to an assessment of needs through social work and should be encouraged to request an assessment [3]. NICE guidelines [6] indicate further that practitioners carrying out or contributing to carer assessments should ensure that: a) the assessment covers all aspects of health wellbeing and social care needs; b) details are shared with other practitioners who are involved in the assessment; and c) those who are carrying out assessments are trained to do so.

### Guidance on signposting of carers

**Fig. 3:**
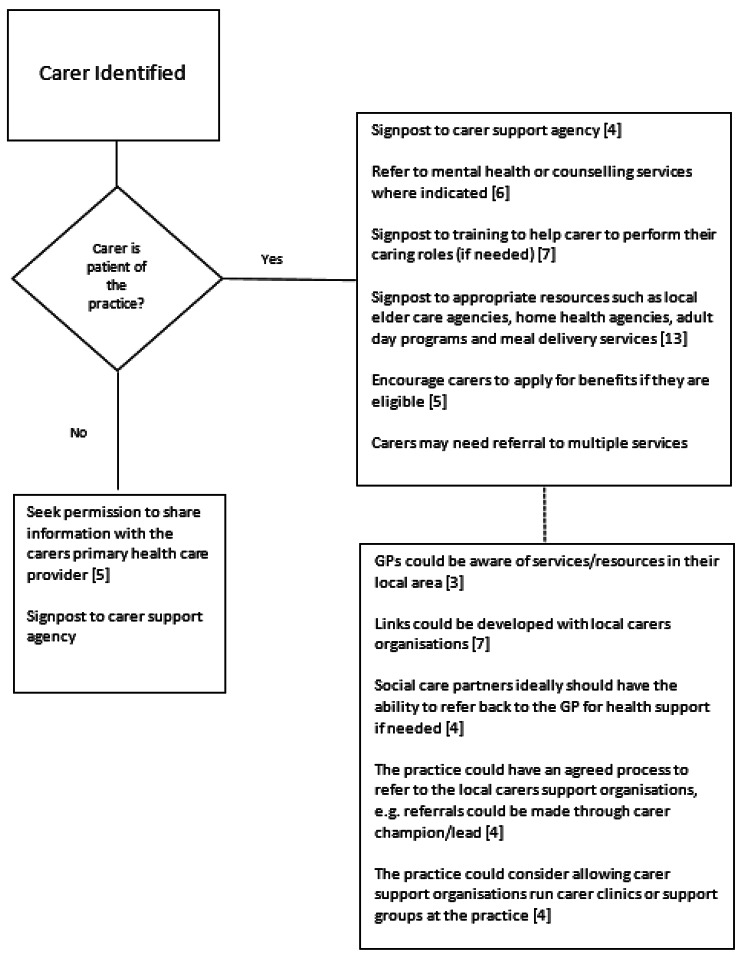
Summary of signposting/referral routes

Seventeen of the included studies/guidelines mentioned referral or signposting of carers to supports. Nine guidelines/best practice papers offer recommendations on referral/signposting [1, 3–7, 11, 13, 57] (see Fig. 3). For example, these suggest referral to community resources, counselling, and training. NHS England, in outlining an integrated approach to identifying and supporting carers (principle 3, p.16), indicate that carers should be encouraged to access appropriate services, with referral to carer support services as possibly the best way by which this may be achieved [4]. According to Sunne (2017), referral to supports, such as carer support agencies, may be achieved through an appointed team member who is the primary contact for patients and families [46]. These agencies or social care partners, in turn, should have the ability to refer back to the GP for health support, if needed [58]. However, a key barrier to meaningful consultation and referral for support exists when a carer is not a patient of the practice, even if the care recipient is already registered there [55]. In this instance, it has been suggested in a guideline for doctors in British Columbia, that the GP could consider offering to write to the carer’s GP regarding their caring role [5].

The RCGP, in their action guide for GPs, provide details of national carer charities, government websites (e.g. NHS Carers Direct, Directgov), helplines and carer support projects [8] to which GPs can refer carers. In a follow-up summary report [7], they describe exemplar practices where staff had developed good relationships with their local carer support organisations. There were some differences in these practices with regard to the ways in which carers were signposted to supports; for example, some were referred through their own carer registration form, while others used a referral form provided by the local carer support organisations. Two guideline documents included a specific focus on mental health referrals [1, 57], particularly with regard to bereavement [57], and although NICE guidelines acknowledge that no evidence regarding referral pathways is currently available, they recommend that a referral should be made to appropriate services in the case of an identified mental health problem [6] (p.27).

### Resources for GPs/carers

Several guidelines provided links to services and resources. For example, “Carers and Young Carers: A GP Resource”, published by RCGP Scotland, provides a comprehensive list of carer support services [3]. Likewise, “Caregiver Care”, an ‘American Family Physician’ publication, provides a list of caregiver resources, including tools such as the AARPs (formerly the American Association of Retired Persons) ‘Prepare to Care’ guide for carers that can be accessed online and provided to a carer during the consultation. They also include online resources, websites, and apps that may be of use to GPs [13]. In British Columbia, a community resource for family caregivers is available, including links to support agencies, as well as details of financial benefits for family caregivers [5]. Robust systems for referring carers are also part of the NHS quality markers for supporting carers in general practice, which refer specifically to, for example, the ability of the practice to refer to local carer support organisations, whether there is an agreed process in place for this referral, and if the practice allows carer support organisations to run carer clinics or support groups at the practice [58].

Parmer [29] developed a set of six health workforce training competencies for HCPs encountering family carers. One of these, entitled ‘Competency E’, refers to “navigating the health and social systems and accessing resources” (p.5) and recommends that referrals to other providers, in line with the family carer preferences, should be part of health care workforce training. Although referral to other agencies was more common, ten of the included studies also mentioned, or contained, resources for GPs to assist them in their consultations with family carers. A summary of resources identified from this review, is provided in Table 3.

**Table 3:** Resources to support GPs and carers

**Resources to support GPs in their role with carers**
Carer Assessment tool (s) [2, 13, 26, 44, 48, 51–53]
URL Links to online resources [13]
Information on resources related to caregiver support agencies, education resources, online resources, bereavement helplines, community-based health services, condition-specific supports and hospice [5].
Information sheet on financial benefits for family caregivers as part of toolkit/GP Resource [5]
Links to examples of where practices had implemented carer support [14]
Information on technology or Apps that can support carers in their role [13]
Link to carer resource page available on GP professional institute [3]
Resources to support practices to implement recommendations [14]: A step-by-step guide to developing a practice action plan A self-assessment checklist for auditing how a practice supports carers.
**Resources to support Carers (to be given by GP or practice)**
Carer Information pack [14]
Handout/pamphlet/leaflet for carers covering [13]: Who is considered a caregiver? What the benefits and challenges to caregiving are What the doctor can do to help How carers can help themselves Where more information can be found
A letter explaining the how the practice can support them [24]
‘Who to call’ fridge magnet with useful numbers [24] (for those approaching end of life)

### Level of evidence

The level of evidence for the included research studies was low, overall (Table 2), with 89% of the studies rated falling in level VI category, and only one study each at level I and level II. No evaluation of the guidelines was reported. The NICE paper “Supporting Adult Carers”, included details on the evidence that was reviewed in the development of their guideline [6]. Other guidelines, for example, ‘Think Patient, Think Carer’, from the Northern Sydney Health District, report that they drew on evidence from the UK paper, ‘Supporting carers, an action guide for GPs and their teams’. ‘Carers Trust Wales’ report that academic and other sources of information were consulted in the development of their guide. Formal audit tools to evaluate the guidelines provided to GPs, were not evident in the review, apart from the Royal College of General Practitioners (RCGP); its action guide for GPs, produced in partnership with the Prince’s Trust for Carers, includes an audit tool called the ‘RCGP Self-Assessment Checklist’, that can be used to determine if a practice is adequately supporting carers [8] (p.35). Additionally, the National Health Service (NHS) provides a set of quality markers to determine if best practice indicators are being met [4].

## Discussion

This scoping review synthesised the available national and international literature on the broad topic of guidelines and good practice standards for GPs to support them in consultations with family carers. Specifically, the review focused on carer identification, assessment, and signposting to supports.

### Carer identification and assessment

The guidelines and research included in the review, suggest that carer identification, widely acknowledged to be a complex process [2, 6], is best achieved as a ‘whole practice’ approach led by the GP and involving other practice staff and appropriate supporting documentation. This finding is in line with previous research by Carduff et al. who piloted an intervention for carers in general practice and found that its success was dependent on whole practice involvement [2]. Our review also identified a range of actions that can be taken by both GPs and practice staff, to encourage carers to self-identify. For example, a key finding in this regard was the appointment of a carer champion or carer lead within a practice. Although our findings outline many of the proposed responsibilities of a carer champion, there is a clear unmet need for more comprehensive information with respect to this role, including selection guidelines, specific role description, accountability, and remuneration (or compensation).

The point of diagnosis was also highlighted as an opportune time to identify carers and has previously been shown to be a time when carers need support [21]. Our review suggests that carer needs should be integrated into care plans developed at the point of diagnosis. Previous literature has indicated there are many barriers to identifying and supporting carers in general practice at policy, practice and health systems level [23]; for example, the carer may not be a patient of the practice [55]. However, a simple solution is highlighted in a guideline produced in British Columbia, which suggests that primary care doctors could offer to write to or refer back to the carer’s own health care provider to inform them of their caring role [5]. However, it is not known how well this would work at a practical level and a need for further research is indicated.

The content of the guidelines was variable, but most offered some recommendations regarding the identification, assessment, and referral of family carers. Notably, most of the included guidelines originated from GP professional bodies. Previous research suggests that GPs are likely to use guidelines more often when they have been developed in collaboration with other GPs and where they have particular relevance to general practice [28]. However, it has also been suggested that GPs are more likely to use guidelines where the content is evidence-based, ideally based on systematic reviews, and where there is transparency regarding the sources of the evidence [27]. The level of evidence for the included research studies was low, overall, and no evaluations of the guidelines were identified. Moreover, only one set of guidelines – the NICE- produced ‘Supporting Adult Carers’ document – was fully transparent, with regard to evidence that was reviewed in its development [6].

The evidence presented here, suggests that consideration should be given to future guideline development, but with a particular focus on transparency and clear and accurate reporting of the existing evidence. Future research might also focus on formally assessing the quality of practice guidelines using an appraisal instrument, such as the Appraisal of Guidelines for Research & Evaluation (AGREE II); this tool has previously been used to assess the quality of guidelines in primary care settings in for example, diabetes management [59] and postpartum care of women and infants [60]. The AGREE II evaluates the quality of guidelines across several domains, including scope, stakeholder involvement, developmental rigor, clarity of presentation, applicability, and editorial independence [61]. However, it should be noted that this tool is not designed to evaluate the uptake or impact of the guidelines in practice or, indeed, the outcomes for family carers. No studies accessing the utility of guidelines in practice and the resultant outcomes for the target population (i.e. family carers), were identified from this review, highlighting an important evidence gap relating to existing guidelines for GPs and the development of audit tools for future guidelines. Furthermore, according to the World Organisation of National Colleges, Academies and Academic Associations of General Practitioners/Family Physicians (WONCA), any future guideline development should ideally incorporate patient and public involvement to ensure that the needs of patients are accurately identified [62].

This review suggests that the assessment of family carers, while recommended, remains an area in need of considerable research and policy development/support. The assessments highlighted in this review were largely relevant to carers from specific caring roles, such as cancer care, end of life, and care of the older person. Conversely, in a recent survey of carers in Ireland (N = 1,484), Family Carers Ireland (FCI) report that the average age of care recipients, was 37 years and that 44% of those surveyed, were caring for a child with additional needs under the age of 18. Thus, a significant gap exists with regard to a generic assessment that applies across caring roles, and that may be effectively used in a general practice setting.

However, the development of a universal carer assessment tool may be challenging due to the different health systems and social policies that exist internationally. For example, in the UK, carers are legally entitled to a carer assessment via their local authority (i.e. the local county council responsible for local health and social care priorities) or voluntary (i.e. not-for-profit) agency [44], a strong policy commitment that does not appear to be replicated in many other countries. It is worth noting that most of the included papers (n = 12) in this review were produced by UK researchers, thereby reflecting a stronger policy imperative in this jurisdiction than elsewhere. Despite this, however, it has been suggested that only one per cent of family carers in the UK are identified through general practice and that, overall, the support of carers is still viewed as secondary within health services [44]. Furthermore, according to a number of UK studies, even when carers do receive an assessment, it often does not lead to any meaningful changes in the support they receive [71, 72]. It is also interesting to note that, further afield, the Australian government has just launched an inquiry into the impact of its Carer Recognition Act on carer outcomes [73]. Thus, current evidence points toward a significant policy-practice gap internationally (to which we have also alluded in our earlier work) [21], which raises questions about the utility of guidelines, albeit these are still needed and an important step in the right direction.

### Signposting and resources

Our previous research suggests that GPs did not have adequate information regarding resources for carers and that this can be a barrier to offering support [21]. This finding has been noted elsewhere; for example, in Australia, a study (N = 66) examining GPs’ awareness of the emotional needs of family carers, highlighted the under-utilisation of community resources within the primary care system mainly because GPs reported difficulties in accessing the required services [74]. The lack of information regarding community resources may be problematic as previous research has highlighted that those carrying out assessments for carers need to have the necessary information regarding where the carer may be signposted for support [44, 55]. Our review has outlined some interesting resources available to GPs, including practical information the GP can offer to carers, such as financial support information, details of carer support agencies or online resources. Arguably, a robust system that allows GPs to access resources in the community requires an approach that involves community support services, such as carer support agencies, reaching out to local primary care/general practices to raise awareness of their services. Equally, an appointed staff member, such as the previously mentioned carer champion, could actively seek out what resources are available in the community.

### Study strengths and limitations

This is the first review, to our knowledge, to scope and synthesise guidelines and recommendations for GPs, with a specific focus on the identification, assessment, and signposting of carers. Whilst previous research examined support for carers in particular settings, such as, cancer care [26], terminal illness [25], and end-of-life (EoL) care [52], this review has identified a comprehensive list of strategies for identifying, assessing and signposting family carers, that may, with more robust empirical evidence, be incorporated into practice and which may be applicable to a diversity of caring roles. This is important, given the increasing and complex care that is provided by both family carers [63] and GPs [64].

We also applied a systematic, transparent, and rigorous methodology [65] coupled with a multidisciplinary team approach [32, 33]. Importantly, whilst the assessment of the quality of individual studies does not normally form part of scoping reviews [31] in clinical settings, we rated the level of evidence for each study in order to increase the transparency around the level of evidence. However, this exercise demonstrated a typically very low level of evidence, highlighting an overall lack of transparency in the field, and furthermore, we were unable to rate the evidence underpinning the guidelines.

Although our review did not seek to identify or synthesis the health risks for carers, the findings provide a selective, albeit not exhaustive, reference list to which GPs may be alerted when a family carer presents at their practice. Information regarding the health risks for carers is also useful in terms of identifying an undisclosed caring role. For example, if a patient presents with these symptoms, the GP may enquire as to whether they are providing care [3]. Importantly, a previously mentioned study by Gallagher et al. [20], indicates that the health risks for carers can persist beyond the cessation of the caring role, yet the mechanisms for supporting former carers did not arise in this review, aside from a recommendation to refer for counselling in the case of bereavement [57]. Future research is needed to determine the requirement for, and parameters of, support for former carers. We consulted with carers in a Public and Patient Involvement (PPI) capacity, to enhance the development of practice guidelines, as recommended by WONCA. Consultation exercises with stakeholders are included in less than 40% of scoping reviews that follow the Arksey and O’Malley framework [66]. However, the current study incorporated both a multidisciplinary team approach to the review and a consultation exercise with carers as primary stakeholders, thereby enhancing the applicability of the findings in clinical practice. The detailed findings from the stakeholder consultation will be reported elsewhere.

Although we conducted a comprehensive search of both peer-reviewed and grey literature, we were limited to papers in the English language due to funding and time constraints. Therefore, some important studies may have been missed. Additionally, in an international context, countries may have differing policy backgrounds in respect of support for carers, particularly in general practice. Therefore, the strategies and approaches identified within this review may be more challenging to implement in certain settings.

## Conclusion

As care needs in our communities continue to increase due to medical advancements and societal and health systems changes, it is becoming increasingly important to put procedures in place to support family carers. Despite considerable evidence indicating that the carer population typically experiences poor physical and mental health due to their caring role, many family carers report that they are rarely, or never, asked about their own wellbeing [21]. GPs, due to their pivotal role in health care [67, 68], are well-positioned to support the needs of family carers. Despite this, very little guidance has been made available to GPs to support them in identifying carers, assessing their needs, and signposting them to appropriate supports. This is problematic because without appropriate guidance and resources, GPs may find it challenging to support family carers, particularly in the context of ever-increasing demands on general practice, such as staff shortages and increasing workload [64]. The findings of the study reported here, add considerable value by identifying models of best practice that may be used to produce high quality clinical guidelines for GPs. We have synthesised data pertaining to the identification, assessment, and signposting of carers to supports, whilst also highlighting a need for health systems and social policies to better support both GPs and family carers in their respective roles.

### Electronic supplementary material

Below is the link to the electronic supplementary material.

Supplementary Material 1

Supplementary Material 2

Supplementary Material 3

## Data Availability

The datasets used and/or analysed during the current study available from the corresponding author on reasonable request.

## Abbreviations

EoL: End of Life
FCI: Family Carers Ireland
GPs: General Practitioners
HCA: Health Care Assistants
HCA: Health Care Professionals
MeSH: Medical Subject Headings
NICE: National Institute for Care Excellence
NHS: National Health Service, UK
OECD: Organisation for Economic Co-operation and Development
RCGP: Royal College of General Practitioners
WONCA: World Organisation of National Colleges, Academies and Academic Associations of General Practitioners/Family Physicians
