# Supplementary material for: Supporting family carers in general practice: a scoping review of clinical guidelines and recommendations
Source: BMC Prim Care. 2023 Nov 6;24:234. doi: 10.1186/s12875-023-02188-1 (PMC10626724; doi:10.1186/s12875-023-02188-1)
Supplement: Supplementary file 2 — Supplementary Material 2 [file 12875_2023_2188_MOESM2_ESM.docx]

**Additional File 2. – Additional information on search strategies and search process**

**1. Framing the research question and formulating search strategies using PICOS**

| **PICOS**  **Framework** | **Broad Areas** | **Specific search terms** | **Inclusion criteria** | **Exclusion criteria** |
| --- | --- | --- | --- | --- |
| **Population** | GPs | general pract* or GP or GPs or family pract* or primary care physician or family physician or family doctor or Medical Home or community care or Family Medicine or Primary Health Care or ambulatory care or Community Care | GPs working in general practice or primary care | Condition specific specialists |
|  | Family Carers | carer* or caregiver* (spouse or son or daughter or unpaid or informal or lay) or care-giver*. | Those providing upaid care to one or more family members/partners/loved ones | Paid carers in healthcare setting such as nursing homes |
| **Intervention** | GP Guidance  Identify/Refer/Assessment | best practice* or guid* or polic* or procedure* or algorithm* or protocol* or position paper* or standard* or white paper* or consensus statement or toolkit or consensus or practice recommendation* or pathway* or model or bundle or checklist* or recogni$* or refer* or assess* or screen* or signpost* or health impact assessment* or need* assessment* or social prescri* or community prescri* or community refer* or social refer* or social intervention or intervention or scheme*) | Interventions/  guidance that support/encourage the identification of carers by GPs  Interventions/  guidance that supports the health and welbeing of the carer | Studies validating assessments / testing psychometric properties  Interventions that support carers in other settings such as voluntary sector or condition specific services |
| **Comparison** |  | No specific search terms |  |  |
| **Outcome** | Support for identification, health and wellbeing assessment and signposting of carers in general practice | No specific search terms |  |  |
| **Setting** | Support for family carers health and wellbeing in general practice setting | Included in GP terms | Settings in General Practice/  Primary Care | Hospital or nursing home setting  Other healthcare settings |

**2. Database search strategies.**

OVID MedLine: Searched 9^th^ November, 2020

| (general pract* or GP or GPs or family pract* or primary care physician or family physician or family doctor or Medical Home or community care or Family Medicine or Primary Health Care or ambulatory care or Community Care).tw. |
| --- |
| *General Practitioners/ |
| *Primary Health Care/ |
| (((carer* or caregiver*) adj3 (spouse or son or daughter or unpaid or informal or lay)) or carer* or caregiver* or care-giver*).tw. |
| *Caregivers/ |
| *Needs Assessment/ |
| *social support/ |
| exp *Burnout, Psychological/ |
| exp Psychosocial Support Systems/ |
| (best practice* or guid* or polic* or procedure* or algorithm* or protocol* or position paper* or standard* or white paper* or consensus statement or toolkit or consensus or practice recommendation* or pathway* or model or bundle or checklist* or recogni$* or refer* or assess* or screen* or signpost* or health impact assessment* or need* assessment* or social prescri* or community prescri* or community refer* or social refer* or social intervention or intervention or scheme*).tw. |
| *practice guideline/ |
| (Policy and procedure manuals).mp. [mp=title, abstract, original title, name of substance word, subject heading word, floating sub-heading word, keyword heading word, organism supplementary concept word, protocol supplementary concept word, rare disease supplementary concept word, unique identifier, synonyms] |
| *Health Policy/ |
| (referral and consultation).mp. [mp=title, abstract, original title, name of substance word, subject heading word, floating sub-heading word, keyword heading word, organism supplementary concept word, protocol supplementary concept word, rare disease supplementary concept word, unique identifier, synonyms] |
| *"Referral and Consultation"/ |
| *Patient Identification Systems/ |
| Decision Trees/ |

**3. Searching of GP professional bodies.**

| Country | Website searched |
| --- | --- |
| Netherlands | <https://www.nhg.org/english/about-us> |
| USA | <https://aagp-academy.org> |
| Canada | <https://policybase.cma.ca/permalink/policy11898?_ga=2.211452435.742776015.1605211635-1091635014.1605211635> |
| New Zealand | <https://gpnz.org.nz/page/2/>  <https://www.rnzcgp.org.nz/RNZCGP/Publications/RNZCGP/Publications/Publications.aspx?hkey=691f9c48-a72e-46d5-8f48-3a345b996252> |
| Australia | <https://www.racgp.org.au/clinical-resources/clinical-guidelines/guidelines-by-topic/view-all-guidelines-by-topic> |
| Belgium | : <https://www.woncaeurope.org/institutes/display/4f30823d-2b9c-466d-86ee-8c21c29d1136/Belgian-Society-for-General-Practitioners-Family-Physicians> |
| UK | <https://www.rcgp.org.uk> |
| Sweden | <https://sfam.se/foreningen/organisation/in-english/#:~:text=SFAM%20is%20the%20professional%20and,organisation%20with%20about%202000%20members>. |
