# Supplementary material for: Supporting family carers in general practice: a scoping review of clinical guidelines and recommendations
Source: BMC Prim Care. 2023 Nov 6;24:234. doi: 10.1186/s12875-023-02188-1 (PMC10626724; doi:10.1186/s12875-023-02188-1)
Supplement: Supplementary file 3 — Supplementary Material 3 [file 12875_2023_2188_MOESM3_ESM.doc]

**Additional File 3**

**Scoping Review – Details of database searches**

| Source | Platform | Date of Search | Filter applied | Records retrieved |
| --- | --- | --- | --- | --- |
| 1. MEDLINE | Ovid SP | 9^th^ Nov, 2020 | 2010 – 2020  English | 1192 |
|  |  |  |  |  |
| 2. NICE | Nice.org.uk | 28/09/2020 | N/A | 169 |
|  |  |  |  |  |
| 3. COCHRANE | Cochranelibrary.com | 9^th^ Nov, 2020 | N/A | 22 |
|  |  |  |  |  |
| 4. PsycINFO | ESBCO | 9^th^ Nov, 2020 | 2010 – 2020  English | 480 |
|  |  |  |  |  |
| 5. CINAHL | ESBCO | 9^th^ Nov, 2020 | 2010-2020 | 2,454 |
|  |  |  |  |  |
| 6. GOOGLE SCHOLAR | Googlescholar.com - 1^st^ 200 | 27/10/20 | 1^st^ 200 | 200 |
|  |  |  |  |  |
| 7. LENUS | Lenus.ie | 27/10/20 | 2010 – 2020 | 74 |
|  |  |  |  |  |
| 8. OpenGrey | Opengrey.eu | 28/10/20 | N/A | 7 |
|  |  |  |  |  |
| 9. Other (government wesites /professional bodies) | See additional File 2. | 10/11/20 | N/A | 0 |
|  |  |  |  |  |
| 10. Kingsfund | Kingsfund.org.uk | 2/11/20 | 2010 - 2020 | 47 |
|  |  |  |  |  |
| 11. Hand searching papers | N/A | 15/12/20 |  | 6 |
| TOTAL before de-duplication |  |  |  | 4,651 |
| TOTAL after de-duplication |  |  |  | 4,430 |
| Removed after Initial screening of title and abstract | | | | 4,461 |
| Imported into Rayyan | | | | 69 |
